# Changes in antioxidant and biochemical activities in castor oil-coated *Capsicum annuum* L. during postharvest storage

**DOI:** 10.1007/s13205-018-1284-1

**Published:** 2018-06-01

**Authors:** Jitendriya Panigrahi, Mansi Patel, Niyati Patel, Bhumi Gheewala, Saikat Gantait

**Affiliations:** 1 Department of Biotechnology, Shri A. N. Patel Post Graduate Institute of Science and Research, Anand, Gujarat 388001 India; 2 All India Coordinated Research Project on Groundnut, Directorate of Research, Bidhan Chandra Krishi Viswavidyalaya, Nadia, Kalyani, West Bengal 741235 India; 3 Department of Genetics and Plant Breeding, Faculty of Agriculture, Bidhan Chandra Krishi Viswavidyalaya, Nadia, Mohanpur, West Bengal 741252 India

**Keywords:** Antioxidant activity, Castor oil, Green chilli, Postharvest, Storage-life

## Abstract

This study, for the first time, evaluates the efficiency of castor oil when used as an external coating on *Capsicum annuum* L., to increase postharvest storage-life at 4 ± 1 °C. The castor oil-coated fruits were successfully stored for 36 days, while the non-coated fruits could only sustain for 18 days. Throughout the storage period (at 9-day intervals), different antioxidants and biochemical assays (allied with storage) such as titratable acidity, ascorbic acid content, ferrous ion chelating activity, reducing power, DPPH scavenging activity, hydroxyl radical scavenging activity, total phenolic content, total sugar estimation, and enzymatic study of polyphenol oxidase and pectate lyase, were assessed. During storage, the castor oil-coated fruits showed a substantial decrease in titratable acidity, ascorbic acid content, total phenolic content, including antioxidant activities such as reducing power and DPPH activity; however, an increase in ferrous ion chelating activity, total soluble sugar content, polyphenol oxidase activity and initial pectate lyase activity was observed, in contrast to that of the non-coated fruits. The application of castor oil proved to be effective in delaying the ripening process of fruits during storage.

## Introduction

*Capsicum annuum* L. (green chilli), an important member of Solanaceae family, is mainly grown as a cash crop owing to its distinctly pungent non-climacteric fruits, enriched with vitamins and minerals. Inspite of having such high nutritional values it has limited storage-life (Panigrahi et al. 2017), which is a major problem for fruits and vegetable growers. Following its harvest, the fruits undergo gradual deterioration due to desiccation, oxidative reactions, microbial growth, and other biochemical changes. Such rapid deterioration in the form of softening, wrinkling, wilting and decaying of the harvested produce during storage and transportation, fail to provide the anticipated profit, consumer-acceptance or to even meet the actual cultivation cost. Softening of fruits is directly linked with water loss during storage (Lownds et al. 1994; Rao and Shivashankara 2015).

Herein, external coating(s), acting as partial barrier to water vapour, gases and restricting rapid metabolism, might be helpful to preserve the texture, quality and external appearance of harversted fruits, and in due course may also improve their storage-life, significantly influencing the functions of storage-associated biochemicals and antioxidants (Baldwin 1994; Li et al. 2017; Panigrahi et al. 2017). External coatings comprise hydrophobics (lipids or waxes), hydrocolloids (polysaccharides or proteins) or a combination of both the compounds (composite coatings). The coating composition determines the barrier properties of the material with respect to water vapour, oxygen, carbon dioxide and lipid transfer in food systems (Guilbert et al. 1996). Even though hydrophilic coatings function as excellent carbon dioxide and oil barriers and provide strength and structural integrity to the fruits, however, they are not at all effective moisture barriers and can create adverse anaerobic conditions (McHugh and Krochta 1994). Hydrophobic lipid or oil coatings have good water vapour barrier properties, due to their low polarity (Guilbert et al. 1996). Usually, climacteric fruits like green peppers do not show any detectable changes in respiration rates during storage (Conforti and Zinck 2002). However, it was recorded that when cucumber and bell pepper were exogenously coated with chitosan, the rate of respiration noticably reduced (Ghaouth et al. 1991). Furthermore, Wong et al. (1994) reported that when cut apple pieces were treated with multilayer coatings, it aided in maintaining the internal carbon dioxide concentrations.

Castor oil is an important vegetable oil with a rich source of ricinoleic acid, a mono-unsaturated, 18-carbon fatty acid. Ricinoleic acid amid all the other fatty acids, is exceptional since it includes a hydroxyl group on its twelfth carbon; it is for the virtue of this functional group that ricinoleic acid (and castor oil) is more polar than the other fats (Thomas 2012). Castor oil proved to be efficient with its greater resistance to increased CO_2_ and reduced O_2_ in the internal environ of fruits, in comparison to that of the essential oils or mineral oils. Castor oil coating significantly delayed the softening and retained the flavour in fruits (Baldwin 1994). Owing to such property, the castor oil coating on seeds of edible pulse or oilseed crops is being used traditionally, to extend the storage life since long, in the southern districts of Gujarat State, India (Parmar and Jain 2016). There are several reports on postharvest storage-life of *C. annuum* published till date that include semperfresh edible coating (Özden and Bayindirli 2002), shellac-based surface coating (Chitravathi et al. 2014), essential oil (cinnamon) coating (Ali et al. 2014), and gibberellic acid coating (Panigrahi et al. 2017).

However, there is no report that addresses the efficacy of castor oil as a coating to slow down the postharvest ripening process till date. In this study, for the first time, we evaluated the influence of castor oil as an external coating on different storage-associated biochemical and antioxidant activities with the aim to extend the postharvest storage-life of *C. annuum* L.

## Materials and methods

### Collection, preparation and castor oil coating of fruits

Mature *C. annuum* fruits (var. New Mexico Chile) were collected from the local market. The collected fruits were thoroughly washed with tap water before dipping in chlorine water for 30 s. After soaking up the adhered water using filter papers, the fruits were drenched in castor oil for 5 s and then wrapped in aluminium foil, prior to their storage in common refrigerator at 4 ± 1 °C. Throughout the storage period (for 36 days with the intervals of 9 days), the following antioxidants and biochemical assays were performed.

### *Titratable acidity* (following Chen et al. 1986)

The fruit pulps weighing 5 g were macerated with 50 ml distilled water and centrifuged at 5000 rpm for 10 min. The supernatant (homogenate) solution was titrated to measure the titratable acidity, using 0.1N NaOH up to a pH level of 8.1.

### *Ascorbic acid content* (following Ranganna 1986)

Ten milliliter of homogenate was mixed with equal volume of 20% meta phosphoric acid, and collected in a 100 ml volumetric flask to adjust the volume with distilled water. The solution was titrated with the standard 2, 6-dichlorophenol indophenol dyes. The ascorbic acid content of each sample was measured by the equation: Ascorbic acid (mg/100 ml homogenate, i.e. 10 g fruit pulp) = (Titre × dye factor × volume made up × 100)/(volume taken for titration × sample weight).

### *Total phenolic content* (following Singleton et al. 1998)

To collect the polyphenol, 1 g of homogenised fruit was mixed well with 10 ml acidic methanol and kept at 4 °C. The solution was then filtered through ordinary filter paper. Next, 150 µl of the above filtrate was mixed with 350 µl of distilled water and later on 2.5 ml Folin Ciocalteu reagent and 2 ml 7.5% (w/v) sodium carbonate were added in it. The solution was then kept in a shaker in the dark for 2 h. The samples were measured at 765 nm with a UV spectrophotometer with gallic acid as a standard. The results were expressed as mg GAE (gallic acid extract)/g extract (fruit).

### *Total soluble sugar content* (following Thimmaiah 2006)

Hundred milligrams of homogenised fruits were mixed with 5 ml of 0.25 N HCl and incubated for a period of 3 h. The mix was then cooled and neutralized by solid Na_2_CO_3_ and the solution volume was adjusted to 100 ml with distilled water. The solution was centrifuged at 2800 g. Next, 1 ml of phenol solution was added in each aliquot. This was followed by the addition of 3 ml of 95% H_2_SO_4_ and incubation for another 20 min in boiling water bath at 25–30 °C. The absorbance was recorded at 490 nm for final estimation.

### *DPPH radical scavenging activity assay* (following Shimada et al. 1992)

A 200 µl of homogenate was mixed with 2.5 ml of methanolic solution of DPPH and incubated for 30 min in dark. The optical density of the solutions was measured at 517 nm, considering methanol with 200 µl of deionized water (to replace homogenate) as blank.

### *Ferrous ion chelating activity* (following Shimada et al. 1992)

The assessment of ferrous (Fe^2+^) ion chelating activity was carried out considering ethylene diamine tetra acetic acid (EDTA) as the control and calculated in terms of regression in absorbance ratio in the presence of polysaccharide and expressed as millimolal (mm) of chelted Fe^2+^ ion.

### *Hydroxyl radical scavenging activity assay* (following Kaur and Halliwell 1994)

The hydroxyl radicals were obtained by a Fenton reaction (Fe^3+^-ascorbate-EDTA- H_2_O_2_ system) and the scavenging activity was measured following deoxyribose method.

### *Reducing power activity assay* (following Benzie and Strain 1996)

A 100 µl of homogenate was mixed with 100 µl of 0.2M potassium phosphate buffer containing 1% potassium ferricyanide. Following incubation at 50 °C for 20 min, 100 µl of 10% TCA, along with 300 µl of distilled water were added in the mixture. Finally, 60 µl of 1% ferric chloride was added and the mixture was incubated for 10 min. Then absorbance was recorded at 700 nm and the recorded absorbance strength was interpreted as reducing power activity.

### *Pectate lyase activity assay* (following Moran et al. 1968)

For the enzyme extraction, 1 g of fruits were homogenized with 15 ml cold NaCl (8.8%) and centrifuged at 13,500 g for 10 min. The supernatant (homogenate) was collected and the pH was adjusted at 7.5 with NaOH. To assess the pectate lyase activity, 2 ml of pectin was mixed with 0.15 ml bromothymol blue and 0.83 ml distilled water. The mixture was incubated at 25 °C in a water bath. The initial absorbance was measured at 620 nm against blank (water). The enzymatic reaction was commenced with the addition of 100 µl enzyme solution and the successive absorbances were recorded at 620 nm up to 80 s at the intervals of 20 s. The unit activity was measured as the quantity of enzyme resulted in 0.01 variation in absorbance.

### *Polyphenol oxidase activity assay* (following Deng et al. 2009)

One gram of fruit pulp was homogenized with 10 ml of 0.05 M potassium phosphate buffer (pH 6.8) and centrifuged at 7000 g for 15 min. The supernatant (homogenate) was treated as enzyme extract. To track the polyphenol oxidase activity, 0.2 ml of the enzymatic extract was reacted with a mixture of 3 ml phosphate buffer and 1 ml 0.02 M catechol. Next, the absorbance values were recorded at 398 nm at an interval of 2 min for final estimation. The results were expressed with the increase in absorbance in every min from each ml of enzyme solution.

### Statistical analysis

All the 10 assessments were arranged in a completely randomized design and carried out in three replications. Twenty samples were used in each replication. Uncoated samples were considered as control. The collected data were statistically analysed by one-way analysis of variance and presented as mean ± standard error that were then compared with each other using Tukey’s test at *P* < 0.05 with the aid of SPSS (version 11, SPSS Inc. Chicago, USA) software.

## Results and discussion

### Titratable acidity

**Fig. 1:**
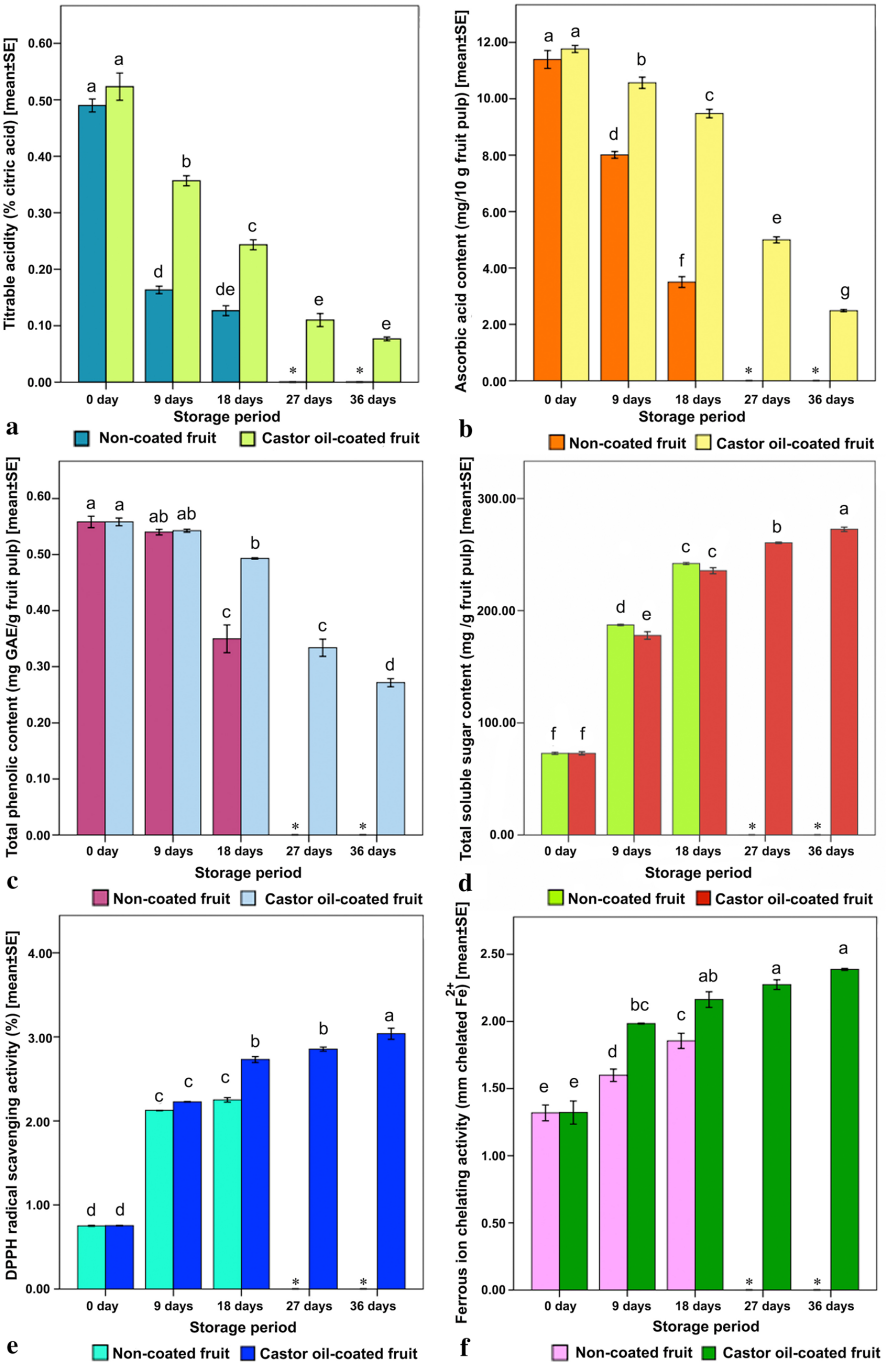
Influence of castor oil coating on titratable acidity, ascorbic acid content, total phenolic content, total soluble sugar content, DPPH radical scavenging activity and ferrous ion chelating activity in *Capsicum annuum* L. during cold storage. Mean columns with different letters are significant at *P* < 0.05 based on one-way analysis of variance followed by Tukey test. *Represents unavailability of information due to postharvest deterioration of fruits

This study shows the potential of castor oil coating in reducing the postharvest losses of storage-life of *C. annuum*. Although the titratable acidity showed a decreasing trend in both non-coated and coated fruits with the passage of storage period, yet at the same time it was better maintained in coated fruits (Fig. 1a). The coated fruits displayed an extended storage-life up to 36 days, which was significantly longer as compared to the non-coated fruits having a limited storage period of 18 days only. On the other note, the decline in titratable acidity value was observed to be instantaneous in comparison to the steady reduction in case of coated fruits. The probable reason of such outcome might be attributed to lesser metabolic activities (Özden and Bayindirli 2002) and delay in consumption of citric acids (Yaman and Bayindirli 2001). Citric acid is the prime substrate for respiration, wherein a decrease in acidity level and a rise in pH level are coupled with highly respiring fruits (Panigrahi et al. 2017). However, the decline in titratable acidity is a vital event during ripening, since it turns the fruits less acidic or sour (Valero and Serrano 2010). Following the harvest of fruits, the respiration increases with a decrease in citric acid and other intermediate products of TCA cycle. The coating on fruit surface might have hindered the sudden rise in respiration and consequent postharvest maturation, which was also reported earlier by Yaman and Bayindirli (2001).

### Ascorbic acid content

The ascorbic acid contents in both castor oil-coated and non-coated fruits showed gradual decline (but with significant difference in between) throughout the storage period. Castor oil coating secured the significantly higher ascorbic acid content (~ 9.476 mg/10 g fruit) at the same storage duration (18 days) than the non-coated ones (~ 3.500 mg/10 g fruit) (Fig. 1b). Similar trend in variation of ascorbic acid content during postharvest storage was also observed in kinnow (Kumar et al. 2000). It has been well established that, ascorbic acid level varies with genotypic variations, pre-harvest climatic conditions, level of maturity and postharvest handling methods (Plaza et al. 2006). Ascorbic acid is generally degraded by oxidative process, which is accelerated in the presence of light, oxygen and enzymes like peroxidase (Plaza et al. 2006). Similarly, during the storage of orange juice it was observed that the vitamin C content was affected by the storage conditions, packing, and processing (Ayhan et al. 2001; Polydera et al. 2003).

### Total phenolic content

Total phenolic content showed an inversely proportional pattern in both the non-coated and castor oil-coated fruits in connection with the storage-duration, yet the quantity was maintained better in the coated ones (Fig. 1c). Immediately after harvest, the phenolic content starts to decline both in non-coated and coated fruits. The total phenolic content at the commencement of storage (at 0 day) was recorded to be the highest (~ 0.558 mg GAE/g fruit). It sustained significantly longer in coated fruits up to a postharvest storage period of 36 days. It was observed that the coated fruits maintained the total phenolic content for a longer period when compared to the non-coated ones. A comparable trend in total phenolic content during postharvest storage of gibberellic acid coated *Capsicum annuum* was most recently reported by Panigrahi et al. (2017). Phenolic compounds are potential antioxidants and free radical scavengers. In addition, phenolic compounds deal with the growth and reproduction, simultaneously protecting the fruits against predators and pathogens (Bravo 2009).

### Total soluble sugar content

With the extension of storage period, the quantity of total soluble sugar tended to increase in both the castor oil-coated and non-coated fruits. Interestingly, the degree of this increase in total soluble sugar content was significantly higher in non-coated fruits at the very early stage (9 days) of storage; however, the coated fruits surpassed the content of total soluble sugar in its later stages of storage (18–36 days). The highest sugar content was observed to be ~ 272.6 mg/g following 36 days of storage in coated fruits. On the contrary, the non-coated fruits registered significantly lower quantities (~ 242.0 mg/g) of sugar even at 18 days of storage, following which the fruits perished (Fig. 1d). As the fruits proceeded towards ripening, the macromolecules degraded into micromolecules in order to be used up rapidly. In contrast of that, in the present study, the mature fruits under storage multiplied the total soluble sugar content and thus maintained their quality for a longer period, that could be highly qualified for consumer acceptance. The present results of change in total soluble sugar content corroborates previous studies on the use of coatings in tomato (Beckles 2012).

### DPPH radical scavenging activity

DPPH radical scavenging activity is recognized based on the power of DPPH (2,2-diphenyl-1-picrylhydrazyl), an unchanging free radical, to bleach out the presence of antioxidants. The castor oil coating on fruits scavenged the DPPH radicals by higher percentages (~ 3.037%) as compared to non-coated ones (~ 2.250%), at the same time it improved the storage-life of fruits up to 36 days (Fig. 1e). The DPPH radical is basically reduced to form DPPH-H and the said reduction is done by polysaccharide extract. A positive correlation of postharvest ripening process and DPPH activity was observed in *Lycium barbarum* fruits (Li et al. 2007). But, initial increase and gradual decrease in the DPPH activity was observed in fresh cut pears and tomato (Oms-Oliu et al. 2008). It is noteworthy to mention that there are also several other factors influencing DPPH activities such as environmental and genetic backgrounds, methodology of harvest and postharvest storage conditions (Dumas et al. 2003).

### Ferrous ion chelating activity

Ferrous ions are most efficient pro-oxidants in biological systems, since their interaction with hydrogen peroxide leads to formation of highly reactive hydroxyl radicals. The ferrous ion chelating activity of coated and non-coated fruits under postharvest storage is shown in Fig. 1f. Significant differences in the trends of increasing ferrous ion chelating activity in coated and non-coated fruits were observed during storage. Ferrous ion chelating activity gradually increased in both the coated and non-coated fruits, but it persisted more in the coated fruits and lasted for 36 days that was substantially (18 days) for a longer duration as compared to the non-coated ones. The role of iron as a transition metal creates free radicals from peroxides by the Fenton reaction and creates several diseases (Halliwell and Gutteridge 1990). Fe^+ 2^ is also used in lipid peroxidation and such reduction of Fe^+ 2^ concentration during Fenton’s reaction can avoid oxidative damage (Singh and Rajini 2004).

### Hydroxyl radical scavenging activity

**Fig. 2:**
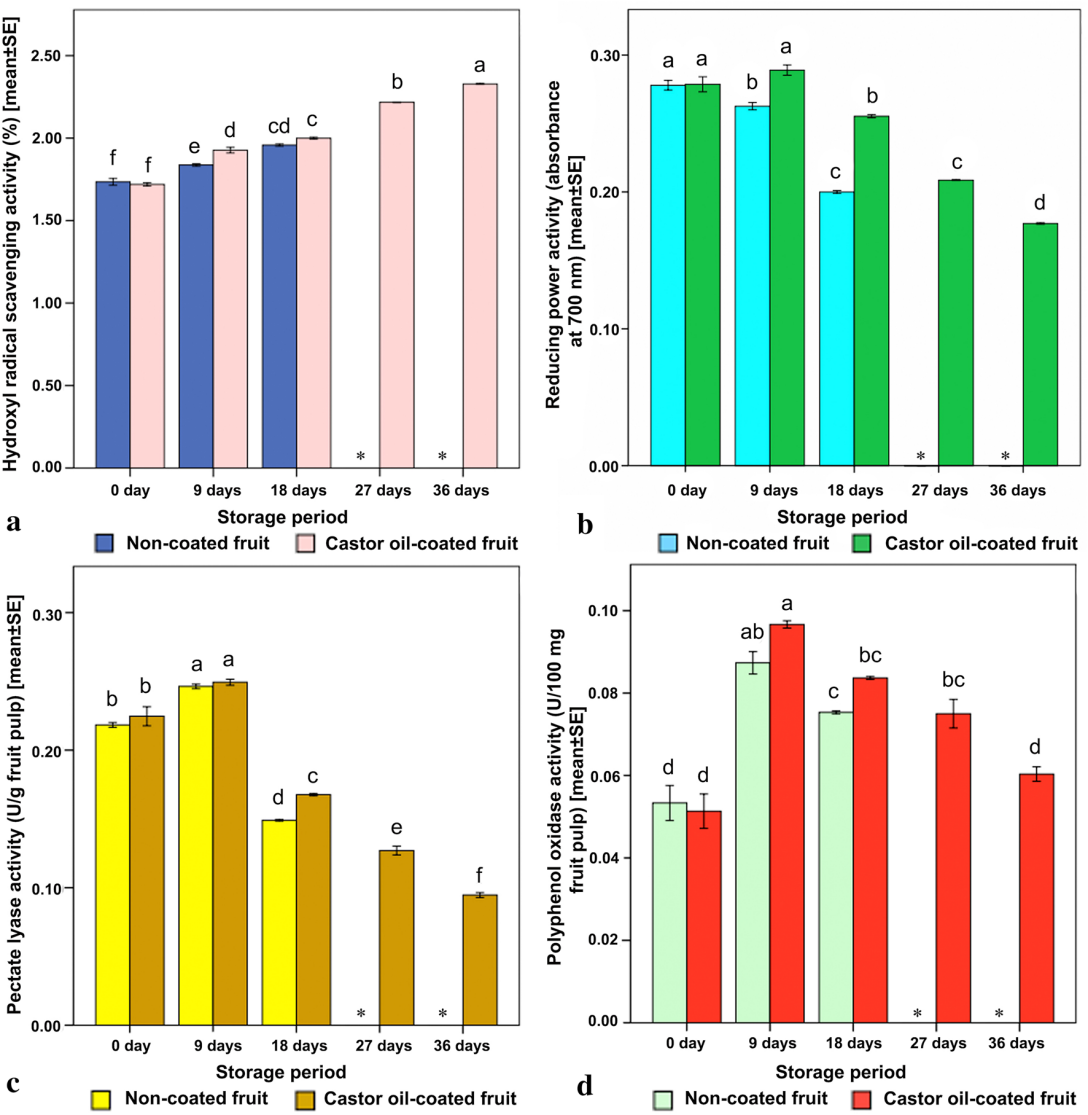
Influence of castor oil coating on hydroxyl radical scavenging activity, reducing power activity, pectate lyase activity and polyphenol oxidase activity in *Capsicum annuum* L. during cold storage. Mean columns with different letters are significant at *P* < 0.05 based on one-way analysis of variance followed by Tukey test. *Represents unavailability of information due to postharvest deterioration of fruits

With the advancement of storage period, the hydroxyl radical scavenging activities of both the castor oil-coated and non-coated fruits were recorded to increase consistantly. However, the coated fruits sustained the 36-day storage period with the highest hydroxyl radical scavenging activity value of ~ 2.324% as compared to the non-coated fruits (~ 2.02%), that thrived only up to a 18-day postharvest storage period (Fig. 2a). The most important active oxygen species that cause lipid oxidation and biological damage are hydroxyl radicals (Gutteridge 1984). In our study, castor oil coating proved to be efficient in minimizing the hydroxyl radical development with enhanced scavenging activity. Similar trend of such increasing hydroxyl radical scavenging activity in gibberellic acid coated *C. annuum* L. was reported by Panigrahi et al. (2017).

### Reducing power activity

The decreased absorbance of reaction mixture represents a drop in reducing power. The reducing power activity of castor oil-coated fruits was recorded to incline at initial stage (9 days) and decline in successive days of storage that by and by extended up to 36 days (Fig. 2b). For non-coated fruits such activity displayed a continuous declining trend. This suggests the direct contribution of antioxidant activity put forward by the castor oil coating during the extended storage of fruits, resisting the aging process. Such initial incline and late decline of the reducing power activity was also reported by Sim and Sil (2008) in *Capsicum* pericarp. Reducing power serves as an important evidence of antioxidant activity. Compounds with reducing power are electron donors and hence can act as primary and secondary antioxidants. Here, during the assessment method, the transformation of the yellow coloured solution to a blue one confirmed the effect on the reducing power of each compound (Dave 2009).

### Pectate lyase activity

Pectic enzymes play the key role to soften the tissues of fruits and vegetables during the ripening process; and it is also during this course that changes in the elements of cell wall and esterification of pectin is typically observed. In our present study, it was observed that the activity of pectate lyase enzyme increased in the beginning of the storage period ~ 0.249 (U/mg proteins) but declined till the end of the storage; this activity lasted (~ 0.095 U/mg proteins) for 36 days in the coated fruits (Fig. 2c) and conversely for 18 days in case of the non-coated fruits. Interestingly, pectate lyase enzyme activity diminished (~ 0.149 U/mg proteins) at a faster rate up to 18 days of storage period in the non-coated fruits. However, the limited activity was observed in all the coated fruits in contrast to that of the non-coated ones, which suggested that castor oil contributed to the formation of a layer over the fruits that might have decreased the availability of CO_2_ and O_2_ and supported natural inhibition of fruit senescence by restricting the functions of cell wall degenerating enzymes. Such restriction in the ripening mechanism through regulated pectate lyase activity was reported in green bell peppers using hydrocolloid-lipid coating (Conforti and Zinck 2002).

### Polyphenol oxidase activity

In the present experiment, the polyphenol oxidase value during initiation of storage was ~ 0.0533 (U/100 mg fruit pulp) (Fig. 2d). The polyphenol oxidase activity showed an initial increase and gradual significant decrease towards the end of the storage period, both in non-coated as well as castor oil-coated fruits. However, the polyphenol oxidase activity comparatively faced greater inhibition in all the coated fruits. Such initial increase and gradual inhibition of polyphenol oxidase activity was reported during cold storage of nitric oxide treated banana. Polyphenol oxidase is a terminal oxidase existing in plants that catalyses oxidation of phenolics resulting in tissue browning of fruits and vegetables (Zhang and Quantick 1997).

## Conclusion

The application of castor oil as an external coating on *C. annuum* fruits resulted in restricted metabolic activities, regulated functions of associated biochemicals and antioxidants during cold storage, delaying the ageing process as a result. Eventually, this has prolonged the storage-life of *C. annuum* up to 36 days. Till date, castor oil is traditionally used as a preservative during storage of food grains (viz. seeds of rice, pulses, oilseeds etc.) only. Based on the results we recorded in this study, it can be suggested that the described method can be used by farmers to preserve other fruits and vegetables for a longer period, since it is non-hazardous, biodegradable and of low-cost in comparison to other chemical coatings.
